# 3-D printing model used to streamline surgical procedures for an intricate condition of airway compression caused by devastating mediastinal chondrosarcoma: a case report

**DOI:** 10.1186/s13256-019-2312-4

**Published:** 2020-01-19

**Authors:** Sen-Ei Shai, Yi-Ling Lai, Hsin-Ni Li, Shih-Chieh Hung

**Affiliations:** 1 Division of Thoracic Surgery, Taichung Veterans General Hospital, 1650 Taiwan Boulevard Sect. 4, Taichung, Taiwan; 2 Institute of Clinical Medicine, National Yang-Ming University, Taipei, Taiwan; 3 The Department of Pathology, Taichung Veterans General Hospital, 1650 Taiwan Boulevard Sect. 4, Taichung, Taiwan; 4 Distinguished Professor & Director, Institute of New Drug Development, China Medical University, Taichung Director, Integrative Stem Cell Center, China Medical University Hospital, Taichung Joint PI, IBMS, Academia Sinica 7F, No. 6, Xueshi Rd., North Dist., Taichung City, 404 Taiwan

**Keywords:** Mediastinal chondrosarcoma, Airway compression, 3-D printing module, T-tube insertion, Thoracoscopy, Radiotherapy

## Abstract

**Background:**

The condition of mediastinal chondrosarcoma causing severe airway compression has never been reported before, and its complexity makes its surgical management challenging. We implemented two new techniques to overcome this problem. Creative mockup analogy of a distorted trachea and tumor lesion using a 3-D printing module, with reprogramming by computed tomography, streamlined the panorama with intricate correlation.

**Case presentation:**

Our patient was a previously healthy 52-year-old slender yellow man who had no obvious medical history. In the last 3 years, upper respiratory tract infection and productive cough were noted frequently, and the patient’s symptoms were aggravated with shortness of breath when his head was positioned below 90 degrees during squatting and hunching of the body. The patient manifested prone sleep with ashen complexion, and he had lost 3–4 kg of body weight over the 3 weeks before admission to our hospital. Virtual bronchoscopy with computed tomography revealed an 8.3 × 7.5 × 4-cm lobulated right upper mediastinal mass with amorphous calcification and severe, intricate airway compression. A creative mockup analogy module of the distorted trachea and tumor was generated by 3-D printing and reprogrammed by computed tomography to streamline the sophisticated correlation. The patient underwent a two-stage operation comprising stabilization of the airway for innovative T-tube insertion preceded by thoracoscopy-assisted radical removal of the tumor. Postoperative adjuvant radiotherapy was administered. The patient recovered uneventfully and stayed healthy for 2 solid years in follow-up.

**Conclusions:**

An advanced 3-D printing model provides affirmative information related to treatment strategy and is also a prospective tool for better doctor–patient communication regarding the disease.

## Background

Chondrosarcoma is a well-defined tumor of firm tissue with calcification. It is more common in males and has unclear pathology. An unusual manifestation of posterior mediastinal tumor included critical airway compression and inaccessible proof before surgical excision. We orchestrated a 3-D printing model portfolio simulated by computed tomography and streamlined the intricate tracheal compression secondary to the devastating tumor, and we propose a valuable tool for precise assessment of complicated tracheal stenosis.

## Case presentation

**Fig. 1:**
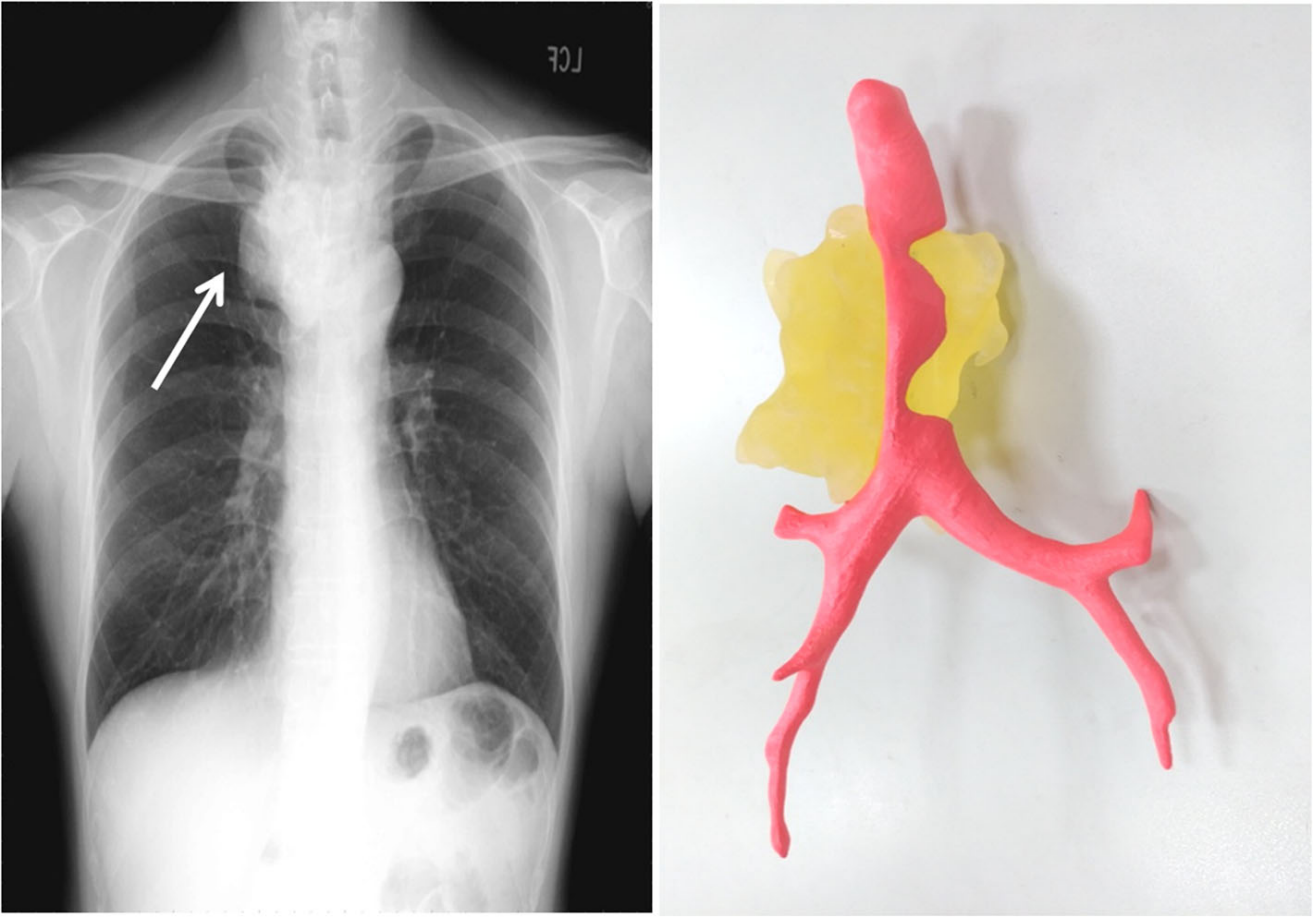
Chest x-ray and the 3-D model of the trachea and chondrosarcoma before surgery. *White arrow* marks the chondrosarcoma

**Fig. 2:**
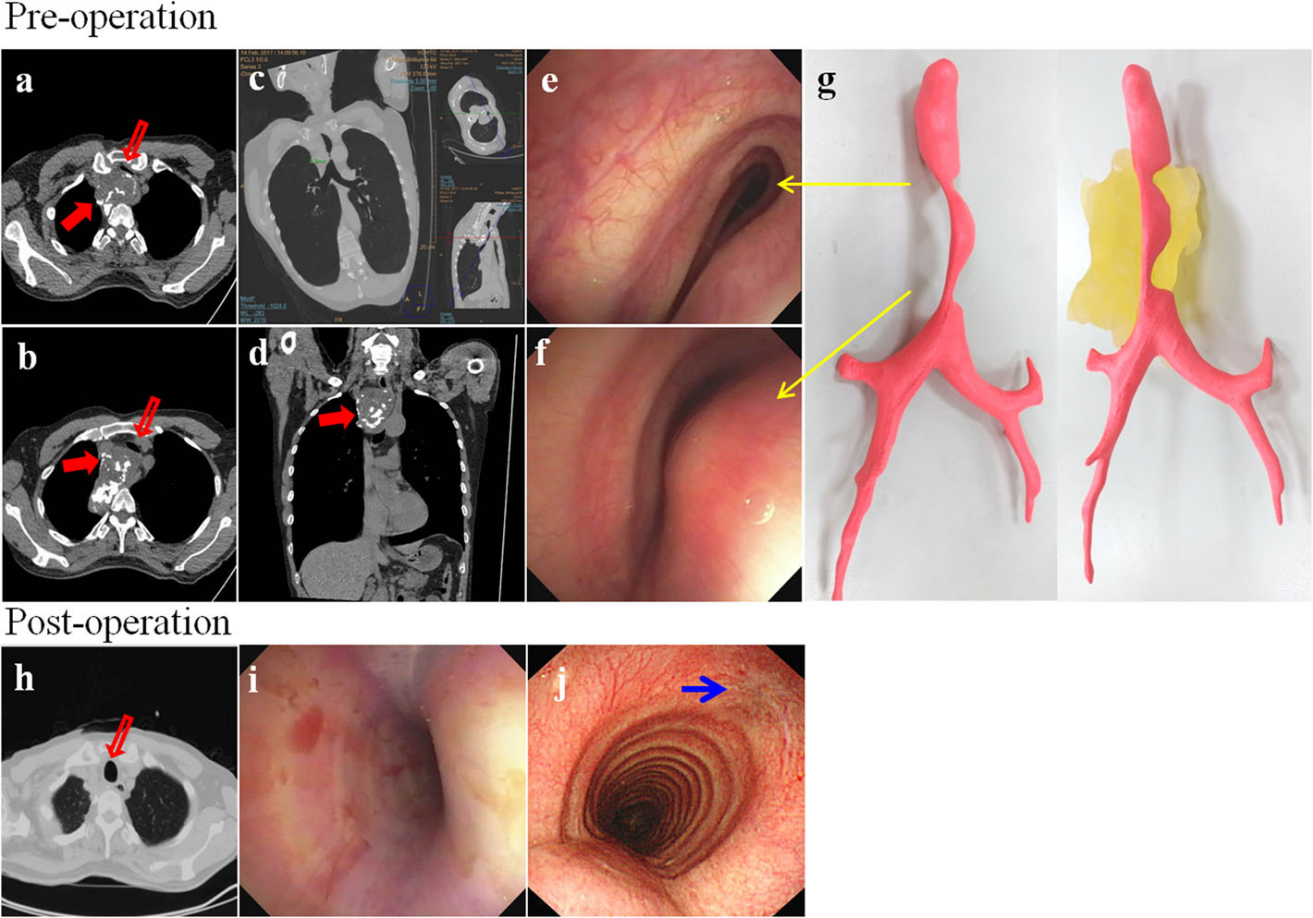
A noncontrast computed tomographic (CT) image shows severe compression of the esophagus and trachea by an approximately 8.3 × 7.5 × 4-cm lobulated tumor with multiple coarse calcifications at right upper mediastinum (**a**–**d**). Endoscopy shows a discrete compressed region of the trachea (**e**, **f**). The 3-D model of the trachea derived from CT images (**g**). The trachea moderately recovered after the insertion of a T-tube stent (**i**). CT scan and endoscopy of the trachea returned to normal after surgery (**h**, **j**). *Empty red arrows* mark the trachea; *solid red arrows* mark the chondrosarcoma; *yellow arrows* mark different sites of the trachea stenosis; *blue arrow* marks the site of tracheostomy

**Fig. 3:**
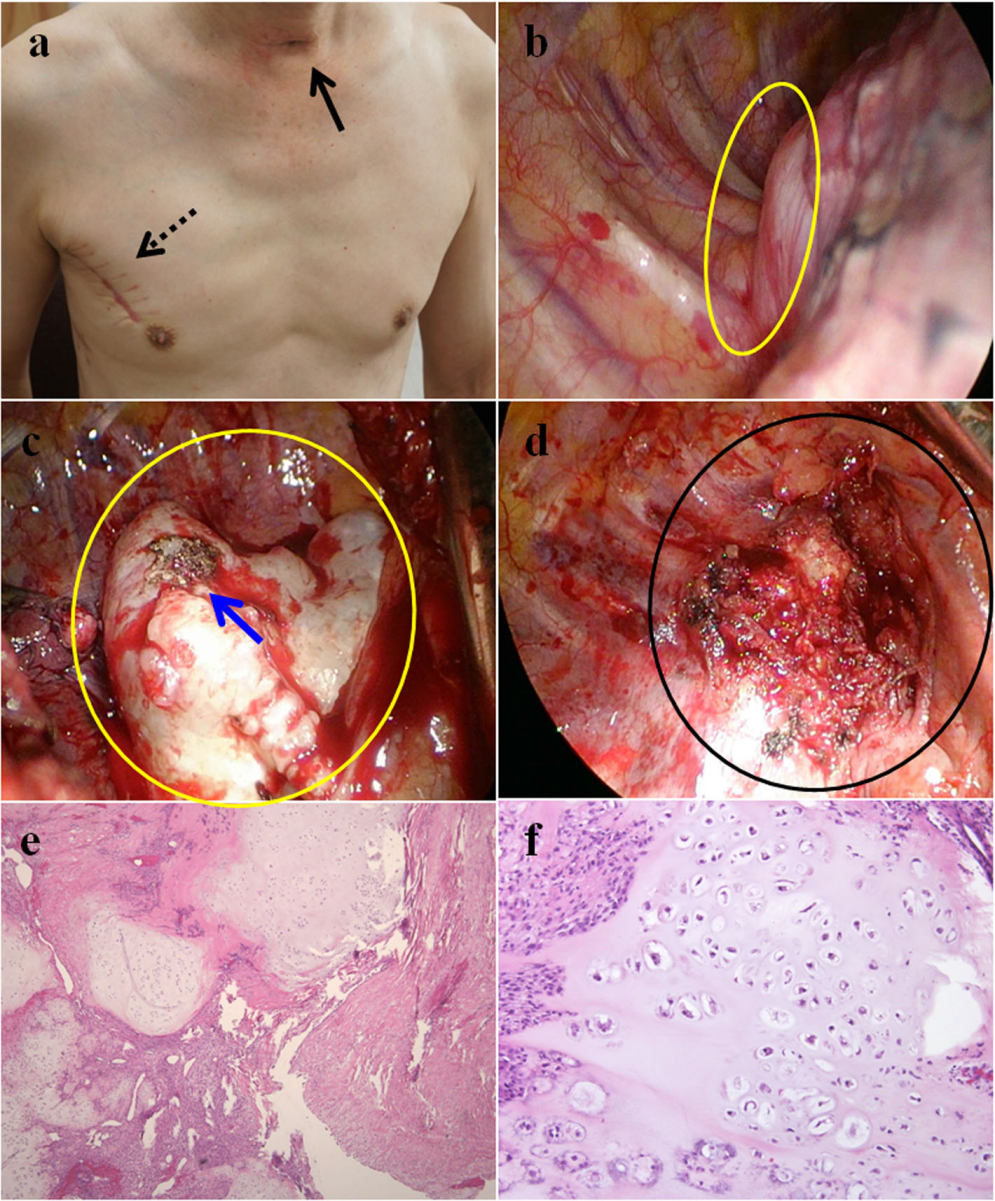
The wound recovered very well after surgery (**a**). Intraoperative photographs show the location of the chondrosarcoma (**b**, **c**) and the view after tumor resection (**d**). H&E staining shows the histology of the chondrosarcoma with low mitotic features (**e**, H&E stain; original magnification × 40; **f**, H&E stain; original magnification × 400). *Black solid arrow* marks the wound of tracheostomy. *Black dotted arrow* marks the surgical wound of 7 cm in size. *Yellow circles* mark the location of the chondrosarcoma. *Blue arrow* marks the site of coagulation. *Black circle* marks the region after the tumor was removed

**Fig. 4:**
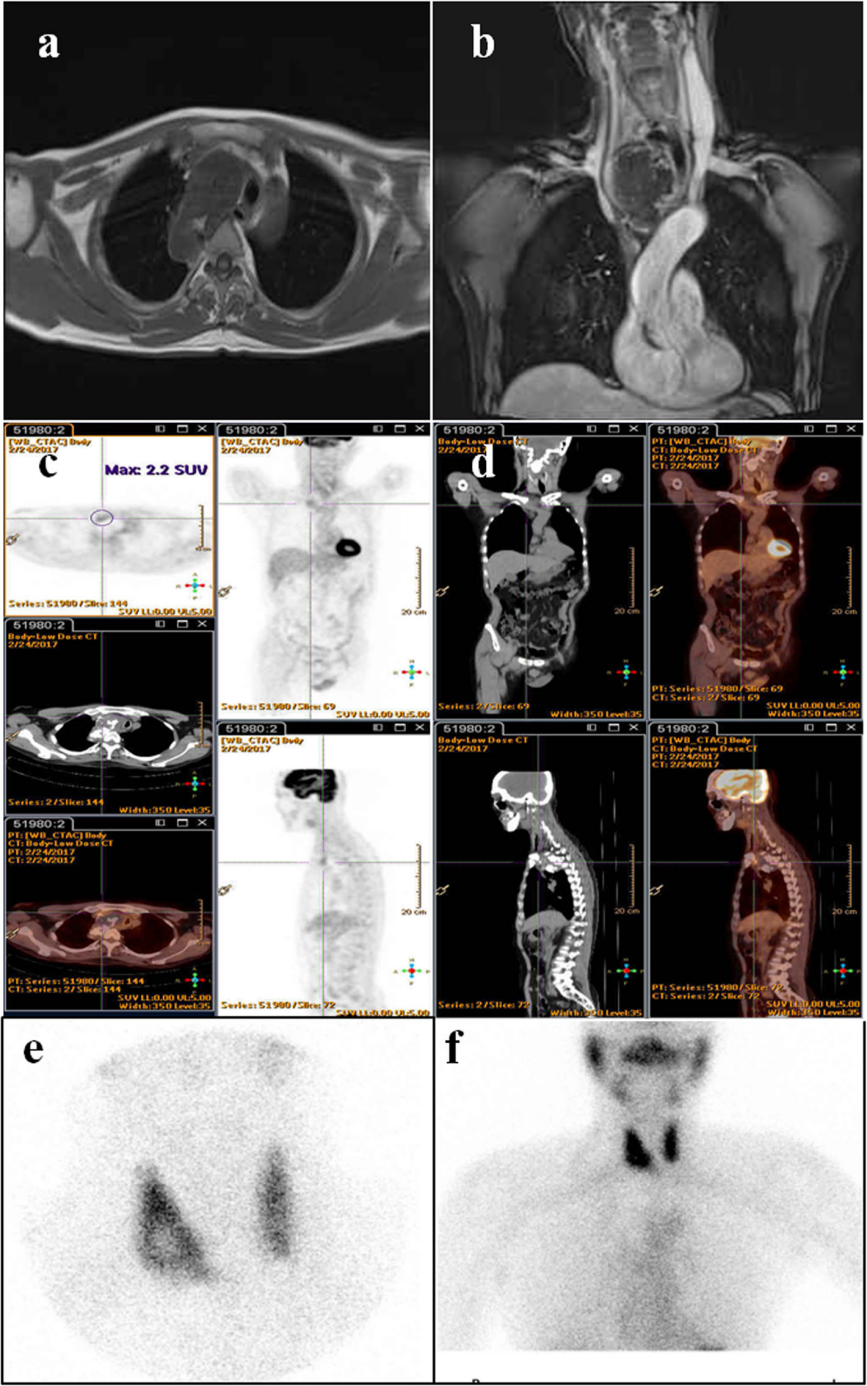
Magnetic resonance angiography demonstrates a hypovascular tumor in the right paratracheal space and compressing the esophagus and trachea. Thoracic angiography shows no significantly abnormal lymph adenopathy in the mediastinum and lower neck and no invasion of greater vessel (**a**, **b**). Whole-body positron emission tomographic scan showing a ^18^F-fluorodeoxyglucose-avid area at the right lower paratracheal region (maximum standardized uptake value, 2.2/1 hour) (**c**, **d**). Tc-99m thyroid scan shows only cold nodule over right lobe of thyroid (**e**, **f**)

Our patient was a previously healthy 52-year-old slender yellow man who had no obvious medical history. About 26 years ago, he was involved in a traffic accident while riding a motorcycle and wearing a helmet, when he was hit by a car in the head. The patient recovered 3 days later, but sequela of severe, stabbing pain was reported between the right medial scapula and spine with radiation to the thorax occasionally thereafter. In the last 3 years, upper respiratory tract infection and productive cough were noted frequently, and the patient’s symptoms were aggravated by shortness of breath when his head was positioned below 90 degrees during squatting and hunching of the body. A chest x-ray revealed a 5.6 × 3.9-cm^2^ mass at LMD (Local Medical Department) since then. In the meantime, the patient consulted various medical centers. During the spans of examination and watch, symptoms developed progressively with sharp pain at locations similar to the previous trauma. The patient had dyspnea, which was relieved temporarily by use of a bronchodilator. The patient manifested prone sleep with ashen complexion, and he had lost 3–4 kg of body weight over the 3 weeks before admission to our hospital. Chest x-ray (Fig. 1) and Virtual bronchoscopy with computer tomography (CT) revealed an 8.3 × 7.5 × 4-cm lobulated right upper mediastinal mass with amorphous calcification and severe, intricate airway compression (Fig. 2a–d). A creative mockup analogy module of the distorted trachea and tumor was generated by 3-D printing and reprogrammed by CT scan to streamline the sophisticated correlation (Additional file 1: Video S1). Considering the cryptogenic nature of the tumor without the possibility of a percutaneous biopsy, we performed an airway stabilization procedure before performing the operation with the patient under general anesthesia. Initially, we applied local anesthetics to the neck wound with the head left-tilted at a 45-degree lateral decubitus position, and an anterior tracheal ring of 0.6 cm in diameter was removed by cautery. Intraoperative bronchoscopy unraveled a long, twisted segment of life-threatening compression with intact mucosa (Fig. 2e, f). We then used an innovative approach with the patient under general anesthesia to insert a T-tube (13 French, 0.5–7.5 cm, upper and lower arm) assisted by laryngoscopy (Fig. 2i). That allowed smooth procedures for a series of examinations, such as magnetic resonance angiography of the thorax, position emission tomography (PET), and thyroid scan (Fig. 4) with preparation for secondary operation of tumor removal. Three weeks later, thoracoscopy-assisted with intralesional surgery of lobulated, firm, gray-tan tumor was performed. The tumor weighed 205 g (Fig. 3b–d). Pathological findings confirmed a low-grade chondrosarcoma with < 1/10 mitotic high-power fields (Fig. 3e, f). The patient recovered uneventfully, and his trachea returned to normal size instantly (Fig. 2h and j) with marked improvement in pulmonary function testing. Postoperative adjuvant radiotherapy was administered, and the patient stayed in healthy condition during a solid 2 years of follow-up.

## Discussion and conclusion

An inaccessible mediastinal tumor causing a long segment of significant tracheal stenosis with synergic hurdles is one of the worst airway crises. The feasible solution of dual formidable devastation overwhelms many experienced surgeons. In our patient, we implemented two innovative techniques to overcome the ordeal. First, we applied the 3-D printing technique for better navigation in the surgical field. The convincing analogy, customized by reprogrammed CT scan of the patient, incarnated the inexplicable substantial to plausible model. Possessing only rudimentary knowledge, the patient and his family could intelligibly visualize modules of the surgical area. The elaborate scheme was carried out in a two-stage operation, with first airway stabilization, followed by tumor resection. A second trick was to resect a tracheal ring with cautery, modified by standard tracheostomy. That resection allowed clear vision of the tracheal lumen for tension-free tube insertion with the patient under local anesthesia. An innovative T-tube insertion temporarily secured the patient’s airway, which bore a hallmark of good voice and breathing, providing an optimal approach for the surgical intervention that followed. The tracheal stoma returned to normal with minimal sequelae after extubation (Fig. 2j).

Posterior mediastinal chondrosarcoma, notorious for local recurrence, is exceedingly rare. In fact, fewer than ten cases have been reported so far, and the condition of compromising the airway has never been published previously in the literature, to our knowledge. The tumor may arise from the tracheal cartilage, vertebral body, or any other cartilage-bearing structure. Predominant features are slow-growing tumor with cartilaginous differentiation [1]. It affects mostly middle-aged men with symptomatic pain [2]. CT is the gold standard for radiographic study [3], typically revealing amorphous calcification scattered in the mass in a punctate, ring, arc, and popcorn patterns. Fine-needle biopsy of the tumor often gives biased samples not representative of the tumor.

Magnetic resonance imaging is useful to depict vascular or neural involvement as complementary information. PET can rule out extrapulmonary metastasis, and standardized uptake value with histological grade may predict postsurgical outcomes. The key to treatment success is early diagnosis of the disease and radical excision. Because extensive resection is impossible with involvement of vital organs nearby, the likelihood of local recurrence remains high after intralesional surgery around the complicated mediastinum. Ozaki *et al.* concluded that a good prognosis in terms of survival can still be expected [4]. Radiotherapy and chemotherapy have limited effects, but radiation therapy can still be considered after incomplete resection in order to achieve better local control [5]. The prognosis for chondrosarcoma is related to its degree of histological differentiation and depends on the quality of the surgical excision. Other prognostic factors have been suggested, such as tumor size and the onset of metastasis. According to some reports, chondrosarcoma affecting the trunk has a prognosis poorer than that affecting the limbs. The advanced 3-D printing model provides affirmative information related to the treatment strategy and is also a prospective tool for better doctor–patient communication regarding the disease.


**Additional file 1: Video S1:** The process of the 3-D model with the ProJet MJP 3600 printer. The data saved as STL files. The available STL files were printed by the ProJet MJP 3600 printer based on the scanned files using the SOLIDWORKS software.


## Data Availability

This case report only contains clinical data from the medical records of the patient reported herein. The data will be made available upon request.

## Abbreviations

CT: computed tomography
PET: position emission tomography
